# Resource partitioning among stranded aquatic mammals from Amazon and Northeastern coast of Brazil revealed through Carbon and Nitrogen Stable Isotopes

**DOI:** 10.1038/s41598-020-69516-8

**Published:** 2020-07-30

**Authors:** Alexandra F. Costa, Silvina Botta, Salvatore Siciliano, Tommaso Giarrizzo

**Affiliations:** 1 Núcleo de Ecologia Aquática e Pesca da Amazônia, Universidade Federal do Pará, Av. Perimetral 2561, Terra Firme, Belém, PA 66040-170 Brazil; 2 Instituto Bicho D’água: Conservação Socioambiental, Travessa B, 183, COHAB, Gleba 2, Marambaia, Belém, PA 66623-311 Brazil; 3 Laboratório de Ecologia e Conservação da Megafauna Marinha, Instituto de Oceanografia, Universidade Federal do Rio Grande - FURG, Rio Grande, RS 96203-900 Brazil; 4 Laboratório de Biodiversidade, Instituto Oswaldo Cruz/Fiocruz, Pavilhão Mourisco, sala 217, Manguinhos, Av. Brasil, 4365 - Manguinhos, Rio de Janeiro, RJ 21040-900 Brazil

**Keywords:** Ecology, Ecology

## Abstract

Aquatic mammals play an important role in community structure. The present study applied stable isotope analysis (SIA) to evidence trophic relationships and resource partitioning among aquatic mammals inhabiting different environments in the Amazon estuarine complex and adjacent coastal zone (AE) and Northeastern coast (NC) of Brazil. In addition, isotopic niche partitioning among *Sotalia guianensis*, *Inia* spp. and *Trichechus inunguis* within the AE was also evaluated, and ecological *S. guianensis* stocks were characterized. Among marine delphinids, the carbon isotopic composition in offshore species mirrored that of nearshore species, contradicting the pattern of decreasing δ^13^C values characteristic of many areas around the world including areas in Southeastern and Southern Brazil. Isotopic niches were highly distinct, with no overlap among the assessed species inhabiting the AE. *Inia* spp. and *T. inunguis* occupied significantly larger isotopic niche spaces, suggesting high habitat plasticity. *S. guianensis* inhabited two coastal regions indicating an ecological distinction. Nitrogen values were similar between *S. guianensis* from the NC and AE, indicating comparable trophic positions. However, NC specimens presented more variable δ^13^C values compared to those from AE. SIA results also allowed for insights concerning habitat use and the trophic ecology of dolphin species inhabiting different oceanographic regions off Northern/Northeast Brazil. These findings provide novel data on the stable isotope composition for cetaceans and sirenians from this region, and aid in furthering knowledge on the trophic ecology and habitat use of the investigated species.

## Introduction

Understanding the ecological roles of aquatic mammals in a given ecosystem is important for the conservation of both the assessed species and their environments. However, information on aquatic mammal trophic ecology and habitat use is generally scarce, mainly due to the intrinsic difficulty of studying these animals in their remote environments. Nevertheless, several studies reported the coexistence and resource partitioning among aquatic mammals in the last few years^1−4^.

Recently, stable isotope analysis (SIA) has become the most common biochemical method used to study aquatic mammal trophic ecology (see^5^). This method is based on the principle that the isotopic composition in consumer tissues reflects the isotopic composition of their assimilated prey^6,7^. Furthermore, nitrogen isotopic values predictably increase from prey to predator^7,8^ and are, thus, applied as consumer trophic position indicators^9−11^. Carbon isotopic values show a lower increase along the food chain, so, they are mainly applied to infer basal sources in their foraging habitats (e.g.^12^). Thus, using this dual isotope approach allows the assessment of the species diet and habitat use which are, in turn, dimensions of its ecological niche^13,14^. Indeed, ecological niches have been described as the “n-dimensional hypervolume”, where dimensions may include both scenopoetic (usually environmental variables) and bionomic (resources) axes^15^. In a similar way, the δ-space formed by the axis δ^13^C and δ^15^N, can be comparable to these scenopoetic and bionomic dimensions of the ecological niche, as isotopic values in a predator’s tissues represent both the prey it consumes (bionomic) and the habitat where it forages (scenopoetic)^13,14,16^. This “isotopic niche” and its dimensions have been recently applied as an approach to study the trophic ecology and resource partitioning of several marine predators, such as baleen whales^17^, toothed cetaceans^2,3,18^, and sharks^19^.

In order to apply SIA to the study of the trophic and spatial ecology of marine predators, the patterns of baseline isotopic values along geographical gradients must be considered. These basal values are commonly used to generate maps of isotopic values (i.e. isoscapes) which in turn provide a powerful approach to understanding the habitat use, foraging ecology and movements of predators^20^ as these baseline differences cascade up, with modifications associated with trophic transfer, to the top of the food webs (e.g.^20,21^. Differences in baseline δ^13^C and δ^15^N values have been reported in particulate organic matter (POM) and plankton along latitudinal and longitudinal gradients in different parts of the world^22−24^. Phytoplankton nitrogen isotopic values are mainly influenced by their nutrient source (i.e. nitrate, ammonium or N_2_), isotopic fractionation during N assimilation and nutrient pool size^25,26^. In general, relatively higher δ^15^N values are found in regions where nitrate is the main source of N for primary producers, while lower δ^15^N values are observed in areas where primary production is mainly supported by N-fixation^25,26^. Basal carbon isotopic values (i.e. phytoplankton δ^13^C), on the other hand, reflect those of the dissolved inorganic C, [CO^2^]aq, temperature, cell size, geometry, and growth rate, and CO_2_ drawdown^27,28^. Nutrient availability also influences phytoplankton δ^13^C values by affecting the growth rate and taxonomic composition of the primary producers^28^. Regions with low nutrient loads have typically low δ^13^C values. Furthermore, the presence of ^13^C-enriched sources of organic carbon (i.e. coastal macrophytes) in coastal regions usually result in a gradient of decreasing δ^13^C basal values towards oceanic waters^20,29^. This typical inshore-offshore gradient of decreasing δ^13^C basal values, however, is not ubiquitous and in regions where large rivers deliver a considerable amount of organic matter to the coastal regions (i.e. Amazon plume), more complex patterns are observed^30−32^. In the Amazon estuary and the marine region under its influence, low δ^13^C values and high δ^15^N values are reported near the estuary and within the most coastal core of the plume, mainly due to the terrestrial origin of the organic matter. Basal carbon isotope values then increase with salinity^31−33^ while the increasing proportion of production based on N-fixation towards oceanic waters generates a decreasing trend in δ^15^N values^32,34^. Nevertheless, the influence of the organic input of the Amazon plume, can reach hundreds of kilometers from the mouth of the estuary, thus influencing the basal isotopic values of large estuarine and marine areas^32,33^.

The Northern/Northeastern Brazilian region, besides the Amazon River Estuary, encompasses a diverse arrangement of habitats, including the longest contiguous mangrove area in the world^35^, with low human population density, reflecting in highly conserved mangroves^36^. In turn, aquatic faunal composition is directly influenced by coastal habitat diversity, fluvial deposit dynamics and river discharge influence^37,38^.

In this region, at least 52 cetacean species^39−42^ and two sirenians^43,44^ have been reported, including several species that may be eligible to be listed as threatened or endangered, but the lack of biological population data hinders the correct evaluation of their conservation status^45−47^. The Northern region is the least surveyed coastal area in Brazil and only two studies present compiled information obtained from stranding and beach surveys^39,48^. Although stranded carcasses are usually a source of biological and ecological data, the high temperatures registered in these tropical regions and the logistical limitations for monitoring these coastal areas usually result in an advanced decomposition state of the carcasses, precluding the access to important biological data, including stomach content analysis, mainly in the case of oceanic species. A better understanding of the trophic and spatial ecology of these aquatic mammals is therefore needed to fill this knowledge gap.

Four cetaceans and two sirenians (Antillean manatee, *Trichechus manatus manatus* and Amazonian manatee, *T. inunguis*) coexist in sympatry in the Amazon estuarine complex, including the Pará River Estuary^49−51^, commonly referred to as Marajó Bay. The cetaceans belong to the Delphinidae (Guiana dolphin *Sotalia guianensis*, and tucuxi *Sotalia fluviatilis*) and Iniidae (Amazon river dolphin, *Inia geoffrensis*, and Araguaian boto *Inia araguaiaensis*) families. Despite decades of surveys, questions regarding habitat use and a possible sympatry between *S. guianensis* and *S. fluviatilis* remain unknown in this region. Recently, the presence of the river dolphins *I. geoffrensis* and the newly described *I. araguaiaensis*^52^ in the Amazon estuarine complex was confirmed through stranding events and molecular analyses^42^.

SIA can lead to important insights into the trophic relationships and foraging habits of these animals. Therefore, in this study carbon and nitrogen isotopes were analyzed in the bone/tooth collagen of aquatic mammals from different habitat use: oceanic (Sperm whale *Physeter macrocephalus*), coastal (e.g., Bottlenose dolphin *Tursiops truncatus*, Rough-toothed dolphin *Steno bredanensis*, Guiana dolphin *S. guianensis*), continental shelf (e.g., Fraser's dolphin *Lagenodelphis hosei*, Risso's dolphin *Grampus griseus*, Melon-headed whale *Peponocephala electra;* Common dolphin *Delphinus* sp*.*) and freshwater/estuarine (Amazon river dolphin *I. geoffrensis*, Araguaian boto *I*. *araguaiaensis*, Amazonian manatee *T. inunguis*). The analysis of low-turnover tissues, such as bone and teeth, can inform on the feeding ecology of an individual over almost its entire lifetime^53,54^, and has, therefore, been widely applied to clarify aspects regarding marine mammal trophic ecology^55−57^.

The main goals herein were to: (1) investigate the habitat use and trophic relationships among freshwater and marine aquatic mammal species, (2) evaluate isotopic niche partitioning among the most representative species (*Sotalia guianensis*, *Inia* spp. and *Trichechus inunguis*) within the Amazon estuarine complex and (3) characterize differences in isotopic niches between ecological *S. guianensis* stocks.

## Material and methods

### Study sites

**Figure 1:**
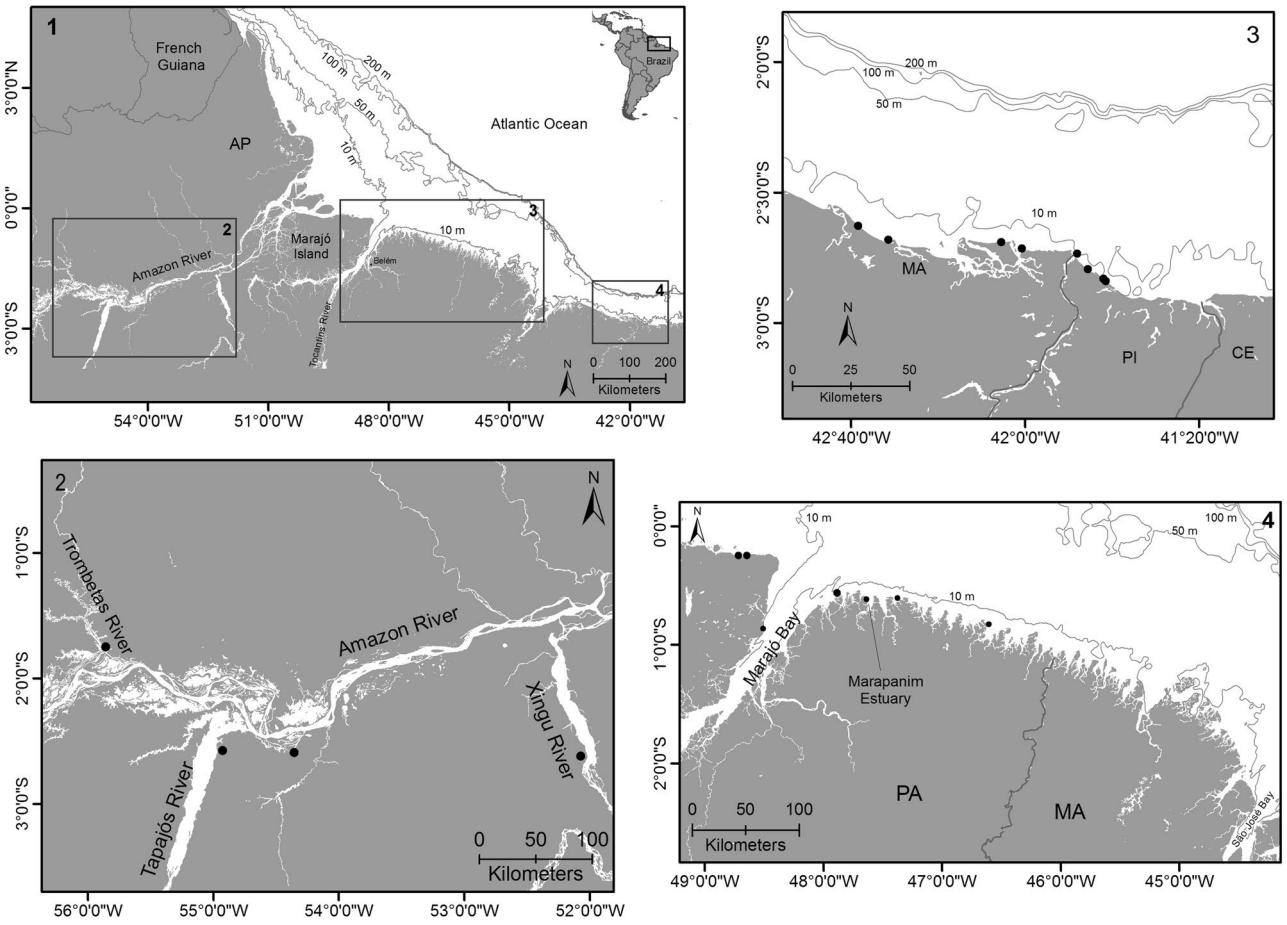
Map of the study sites representing the sampling sectors along the Northern and Northeastern Brazil (1) indicating the 10, 50, 100 and 200 m isobaths. Detailed maps of the sampling regions: (2) Amazon Lowland (AL), (3) Northeastern coast (NC), on the coast of the Maranhão (MA) and Piauí (PI) states, and (4) Amazon Estuary and adjacent coastal zones (AE). Amapá (AP), Pará (PA), Maranhão (MA), Piauí (PI) and Ceará states (CE). Sector AE only includes the study sampling area and not the entire Northern region. Dots indicate the sampling locations.

The samples assessed herein were obtained from stranded carcasses recovered along three main sectors: (1) the Amazon lowland (AL); (2) the Amazon estuarine complex and adjacent coastal zones (AE) and, (3) the Northeastern coast of Brazil (NC) (Fig. 1).

The Amazon lowland (AL) sector comprises the floodplain habitat named *várzea*, consisting of clearwater rivers^58^. Specimens from this region were collected in rivers around the Santarém municipality, Ayaya River, a small Amazon River tributary; at Oriximiná, Trombetas River; at Vitória do Xingu, in the lower Xingu River, and near Belém, Guajará Bay.

The Amazon estuarine complex and adjacent coastal zones (AE) covered in this study belong to the Northern and Eastern coast of Marajó Island (i.e., Marajó Bay), as well as the Eastern coast of the state of Pará. Marajó Bay is formed mainly by Pará discharges and the Araguaia-Tocantins River Basin can be considered as the main freshwater source to this bay^59^. This area receives a superficial saline intrusion during low river discharge^60^ and undergoes a macrotidal regime, with maximum tides around 4 m on both sides of the bay^61^. The continental Amazon shelf is influenced by factors such as proximity to the Equator, strong tides (semidiurnal tides) and oceanic currents and winds (e.g., North Brazil Current, easterly trades), as well as the substantial discharge of the Amazon River (e.g., water, solutes and particulate materials). The region is considered part of the wet tropics, due to high precipitation rates and temperature^62^.

The Northeastern coast (NC) covers the coastline of the state of Piauí and the easternmost coastline of the state of Maranhão. The Parnaíba River discharge into the Atlantic Ocean forms a delta with five tidally influenced bays, Tutóia, Caju, Melancieiras, Canárias and Igaraçu, that form the Parnaíba Delta^63^. The region comprises a mesotidal coast, with tides ranging from 1.1 m to 3.3 m^64^ and vast mangrove forest areas.

### Sample collection

Samples were obtained from floating or stranded carcasses found during sampling surveys or from incidental catches in fishing gear (i.e., *S. guianensis*). Carcasses were recovered regardless of decomposition stage and taken to the *Museu Paraense Emílio Goeldi* (MPEG, Belém, Pará, Brazil) where hard parts (i.e., bones and teeth) were cleaned from outer soft tissues and stored dry. Most samples were collected between 2005 and 2014. However, older specimens of *Inia* spp. and *T. inunguis* collected by naturalists (i.e., Émil August Goeldi and Gottfried Hagmann) and MPEG researchers from the state of Pará (AL) in the 1910s, 1970s and 1980s were also included.

A total of 270 bone and teeth samples from aquatic mammals, representing 14 taxa and four families were assessed, namely Physeteridae (*Physeter macrocephalus*); Delphinidae (*Delphinus* sp., *Globicephala macrorhynchus, Grampus griseus, Lagenodelphis hosei, Peponocephala electra, Pseudorca crassidens, Sotalia guianensis, Stenella attenuata, Steno bredanensis, Tursiops truncatus*), Iniidae (*Inia geoffrensis, I. araguaiaensis*) and Trichechidae (*Trichechus inunguis*).

Most analyzed bone samples were obtained from the internal portion of the skull using pliers. In the absence of a skull, other available bones (*n* = 67) were used, following the priority order: 1st caudal vertebrae, 2nd chevrons, 3rd teeth and other bones (e.g., scapula, mandible, flipper). Tooth fragments were removed after demineralization and lipid extraction was performed for the whole tooth. Previous studies reported a difference of 0.2‰ in carbon and 0.3‰ in nitrogen between the teeth and bones of the same individual^65^. Therefore, the isotopic values of the sampled tissues were considered comparable.

### Stable isotope analysis

Bone fragments were demineralized by repeated baths in hydrochloric acid (HCl, 0.5N for approximately 72 to 96 h^66^ in order to isolate collagen. Each bone fragment (or tooth) was placed in a glass vial covered with acid and stored at 4°C overnight, with acid replacement every 24 h. Samples were subjected to successive baths with distilled water to achieve a neutral pH after a rubber-like flexibility was reached. After this process, lipid extraction was performed by manual rinsing of the samples three times in a 2:1 methanol:chloroform solution, discarding the old solution each time and replacing it with a new one. Samples were then washed in distilled water and dried for at least 24 h at 60°C^56^. Dried samples were then ground to a fine powder using a mortar and pestle. Bone/teeth collagen samples were finally weighed (0.5 to 0.6 mg) in tin capsules (Costech Analytical).

Nitrogen and carbon isotope ratios were measured by Elemental Analyzer Continuous Flow Isotope Ratio Mass Spectrometry in the Center for Stable Isotopes, University of New Mexico using a Costech ECS 4010 Elemental Analyzer coupled to a ThermoFisher Scientific Delta V Advantage mass spectrometer via a CONFLO IV interface. Isotope ratios are reported using the standard delta (δ) notation relative to V-AIR and to Vienna Pee Dee Belemnite (V-PDB), respectively. The three internal laboratory standards are: UNM-CSI Protein std#1, casein purchased from Sigma Aldrich with δ^15^N and δ^13^C values of 6.43‰ and − 26.52‰; UNM-CSI Protein std#2, soy protein purchased from Sigma Aldrich with δ^15^N and δ^13^C values of 0.98‰ and − 25.78‰; UNM-CSI protein Std#4, house made tuna protein with δ^15^N and δ^13^C values of 13.32‰ and − 16.17‰. Analyses were normalized to the laboratory standards which were calibrated against IAEA N1, IAEA N2 and USGS 43 for δ^15^N and NBS 21, NBS 22 and USGS 24 for δ^13^C.

### Data analysis

For *T. inunguis* (*n* = 11) and *Inia* spp. (*n* = 10) we obtained samples from museum specimens of distinct years (1910′s to 2010), therefore, before pooling samples from different decades, possible temporal trends were tested. An ordinary least squares linear trend performed for both taxa didn't detect significant differences in historical trends for *Inia* spp. (δ^13^C: r^2^ = 0.25, *p* = 0.13; δ^15^N: r^2^ = 0.25, *p* = 0.13) and for *T. inunguis* (δ^13^C: r^2^ = 0.06, *p* = 0.45; δ^15^N: r^2^ = 0.24, *p* = 0.12). Before pooling different bones from the same species (i.e., *Sotalia guianensis*, the only species with enough sampling to perform the test: *n* = 220), possible differences in stable isotope signatures were assessed using a permutational multivariate analysis of variance test and no differences were detected (*pseudo*-F = 1.60, *p* = 0.11). So, we assume that for our data set distinct bone tissue of samples didn’t affect results. Due to the small sample size for some species and lack of essential data (i.e., age, standard length, sex), neither sex or maturity stage were considered for the analyses.

δ^13^C and δ^15^N data normality and homogeneity of variance were tested by the Shapiro–Wilk and Levene tests, respectively. The Student’s *t*-test was used to compare isotopic values between *S. guianensis* populations and between marine and freshwater species. A significance level of 0.05 was assumed for all tests.

To investigate isotopic niche variations, species were grouped into Delphinids (all delphinids except *S. guianensis*), *Inia* spp. (*I. geoffrensis* and *I. araguaiaensis*) and *T*. *inunguis. S. guianensis* specimens were grouped according to their sampling sector: Sgui_AE for individuals from AE and Sgui_NC, for specimens from NC. Isotopic niche areas were calculated through standard ellipse areas corrected for small sample sizes (SEAc) using Stable Isotope Bayesian Ellipses (SIBER routine in SIAR package in R^67^). Overlaps among ellipses were also calculated, in order to quantify the trophic overlap among groups. Probabilities were estimated by Bayesian inference indicating uncertainty and central tendency measures based on permutations presenting 50%, 75% e 95% credibility intervals.

## Results

The marine species, exhibited broad ranges of δ^13^C (− 16.6 to − 10.6‰) and δ^15^N (6.6 to 16.0‰) values (Table 1). Significantly higher δ^13^C values (*t*-test, t = 16.0, *p* < 0.0001), but similar δ^15^N values (*t*-test, *t* = 1.9, *p* = 0.06) were observed in marine species compared to species from lowland and estuarine areas.

**Table 1:** Mean and standard deviation (SD) of δ^13^C and δ^15^N isotope values of (in‰) in aquatic mammal bone samples.

Species	δ^13^C					δ^15^N			
	N		Mean ± SD	Min	Max		Mean ± SD	Min	Max
*Trichechus inunguis*	11	–	− 19.6 ± 4.1	− 25.5	− 12.9	–	7.2 ± 0.6	6	8.1
*Inia geoffrensis*	7	–	− 18.7 ± 4.3	− 23.8	− 12.5	–	12.9 ± 1.4	11.6	15.3
*Inia araguaiaensis*	3	–	− 16.5 ± 3.6	− 20.6	− 13.8	–	12.9 ± 0.5	12.6	13.4
*Delphinus* sp.	2	− 12.5	–	–	–	12.1	–	–	–
		− 11.4	–	–	–	12.4	–	–	–
*Globicephala macrorhynchus*	1	–	− 13.1	–	–	–	11.8	–	–
*Lagenodelphis hosei*	1	–	− 13	–	–	–	13.3	–	–
*Grampus griseus*	2	− 11.9	–	–	–	11.5	–	–	–
		− 13.0	–	–	–	14.4	–	–	–
*Peponocephala electra*	2	− 12.6	–	–	–	11.8	–	–	–
		− 12.9	–	–	–	12.1	–	–	–
*Pseudorca crassidens*	1	–	− 11	–	–	–	13.3	–	–
*Sotalia guianensis* (AE)	188	–	− 12.9 ± 1.1	− 16.5	− 11.1	–	11.6 ± 0.9	8.3	13.9
*Sotalia guianensis* (NC)	32	–	− 12.2 ± 0.6	− 13.7	− 10.8	–	11.5 ± 1.0	9.9	13.1
*Stenella attenuata*	2	− 13.1	–	–	–	10.7	–	–	–
		− 13.3	–	–	–	11.2	–	–	–
*Steno bredanensis*	9	–	− 12.0 ± 0.8	− 13.1	− 10.8	–	12.2 ± 1.0	10.8	14
*Tursiops truncatus*	6	–	− 11.3 ± 0.7	− 12.6	− 10.6	–	13.0 ± 1.3	10.5	14
*Physeter macrocephalus*	3	–	− 14.4 ± 1.9	− 16.6	− 12.9	–	15.4 ± 0.6	14.7	16

Specimens were collected from Northern, Amazon lowland (AL), Amazon Estuary and adjacent coastal zones (AE), and Northeastern Brazil (Maranhão and Piauí, NC).

**Figure 2:**
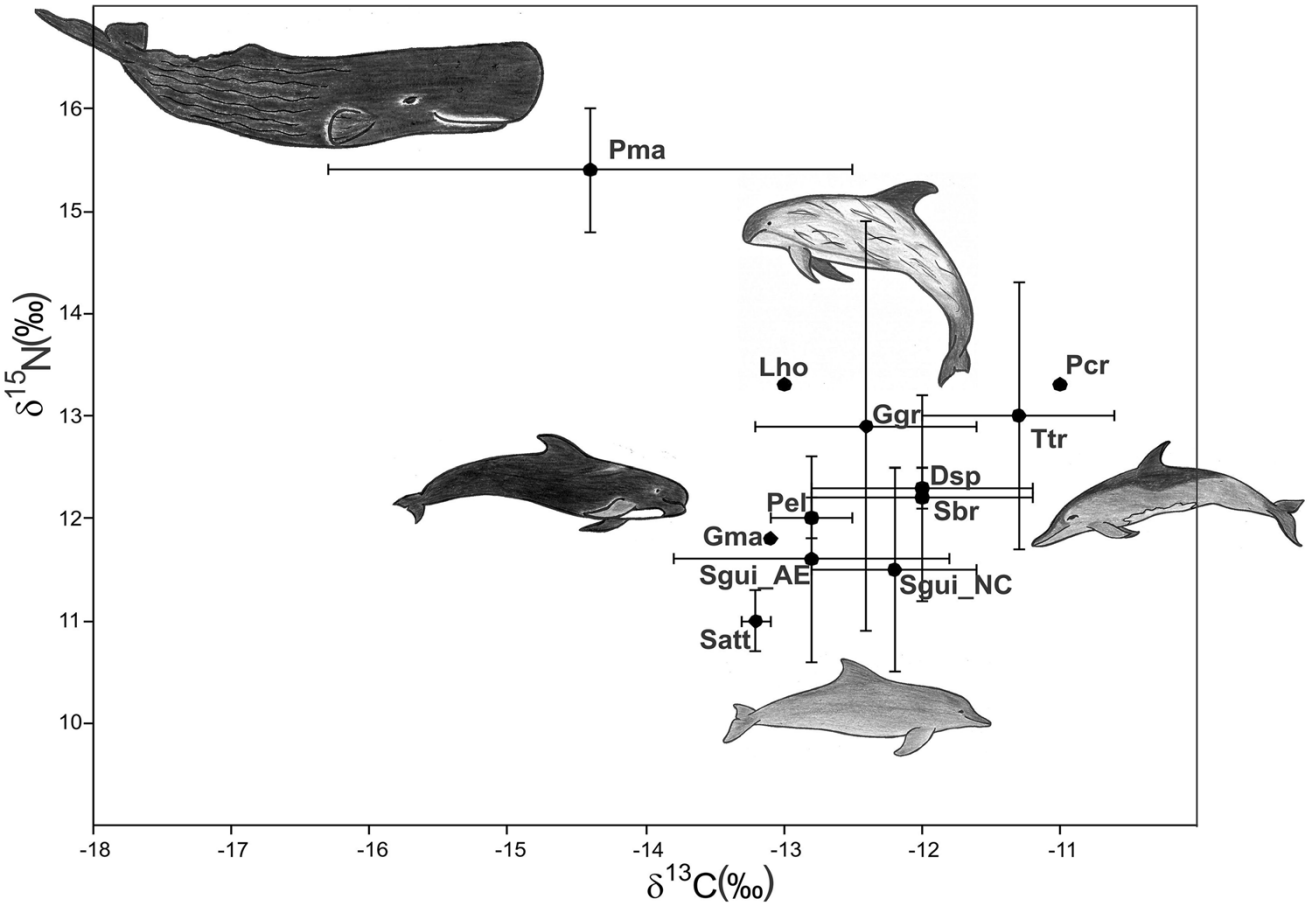
Mean (dots) and standard deviation (bars) carbon and nitrogen isotopic values in bone-collagen of aquatic mammals collected from the Amazon Estuary and adjacent coastal zones (AE) and Northeastern coast of Brazil (NC). Isotope ratios for marine species presented a spatial trend of decreasing δ^13^C values towards offshore habitats for the following delphinids: *Pseudorca crassidens* (Pcr), *Tursiops truncatus* (Ttr), *Delphinus* sp*.* (Dsp), *Steno bredanensis* (Sbr), *Grampus griseus* (Ggr), *Peponocephala electra* (Pel), *Lagenodelphis hosei* (Lho), *Globicephala macrorhynchus* (Gma), *Stenella attenuata* (Sat) and the most depleted in δ^13^C and most enriched δ^15^N of all cetaceans, *Physeter macrocephalus* (Pma). Species drawings are not to scale.

*Sotalia guianensis* from NC presented similar δ^15^N values (*t*-test, *t* = 0.80, *p* = 0.43) but higher mean δ^13^C values than individuals from AE (*t-*test, *t* = − 3.39, *p* < 0.001). Excluding Sgui_AE from the analysis, a pattern of decreasing δ^13^C values was observed from inshore (i.e., *S. bredanensis*, *T. truncatus*) to offshore (*G. macrorhynchus*, *G. griseus*, *L. hosei*, *P. electra*, *S. attenuata*) species. The most ^13^C-depleted values of all marine species were observed for *Physeter macrocephalus* (− 14.4 ± 1.9‰, range − 16.6 to − 12.9‰) and the most ^15^N-enriched δ^15^N values. The only *P. crassidens* specimen analyzed herein presented high δ^13^C and δ^15^N values (Table 1; Fig. 2).

A wide range of δ^13^C (− 25.5 to − 12.5‰) and δ^15^N (6.0 to 15.3‰) isotope values was observed for species that inhabit both AL and AE regions (*T. inunguis* and *Inia* spp.). These species did not differ in δ^13^C values (*t-*test, *t* = 0.86, *p* = 0.40) but differed significantly in δ^15^N values (*t-*test, *t* = 10.4, *p* < 0.0001).

**Figure 3:**
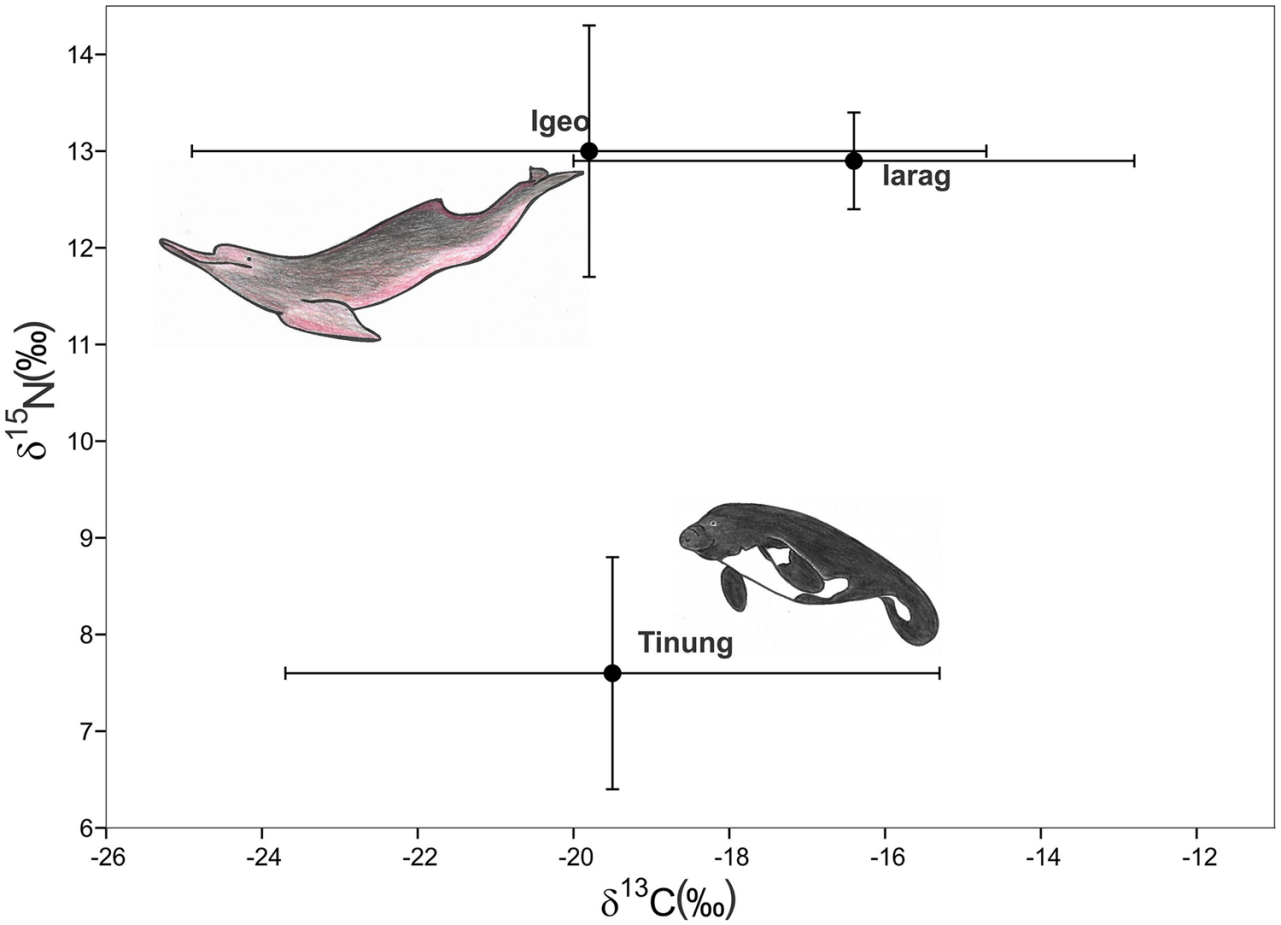
Mean (dots) and standard deviation (bars) carbon and nitrogen isotopic values in bone-collagen for *Inia geoffrensis* (Igeo), *I. araguaiaensis* (Iarag) and *Trichechus inunguis* (Tinung). Species showed significant differences in δ^15^N values but did not differ in δ^13^C values. Specimens were collected along the Amazon lowland (AL), and Amazon Estuary and adjacent coastal zone (AE) in Northern Brazil. Species drawings are not to scale.

*T. inunguis* specimens from AL exhibited a large dispersion of δ^13^C values (− 25.5 to − 15.8‰), whereas those from AE presented a narrow range (− 16.2 to − 12.9‰). *T. inunguis* occupied the lowest isospace position, with δ^15^N values of 7.6 ± 1.2‰ (range 6.0 to 10.3‰) (Fig. 3).

Regarding *Inia* spp., non-significant differences in mean δ^13^C and δ^15^N values were observed between species (Fig. 3, *t*-tests, *t* = 0.77, *p* = 0.46 and *t* = − 0.09, *p* = 0.92, for δ^13^C and δ^15^N, respectively).

### Isotopic niche variation

The isotopic niches were highly distinct among the aquatic mammals that inhabit the AE region (*T. inunguis, S. guianensis*, *I. geoffrensis* and *I*. *araguaiaensis*) with no overlaps (standard ellipse areas, SEAc). In addition, no overlap among the most representative species of the study area (*Sotalia guianensis*, *Inia* spp. and *Trichechus inunguis*) was observed, while a significant overlap among Delphinids and *S. guianensis* populations was noted, expressing isotope niche partitioning.

**Figure 4:**
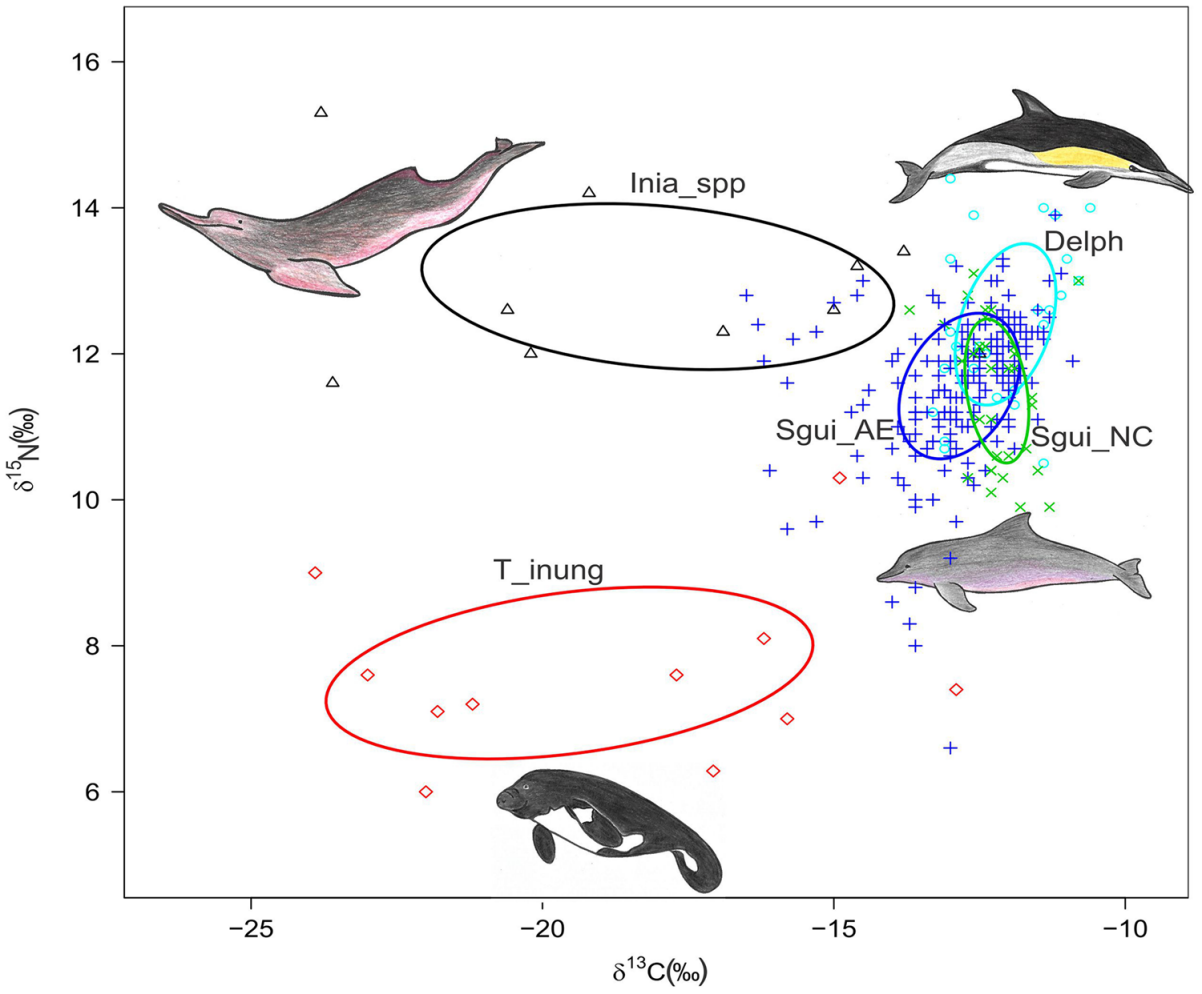
Bayesian standard ellipse areas (solid lines represent SEAc, standard ellipse areas adjusted for small sample sizes), containing c. 40% of data^67^ for Inia_spp (*n* = 10, black line), T_inung (*n* = 11, red line), Sgui_AE (*n* = 188, dark blue line), Sgui_NC (*n* = 32, green line) and Delph (*n* = 26, light blue line). Species drawings are not to scale.

**Figure 5:**
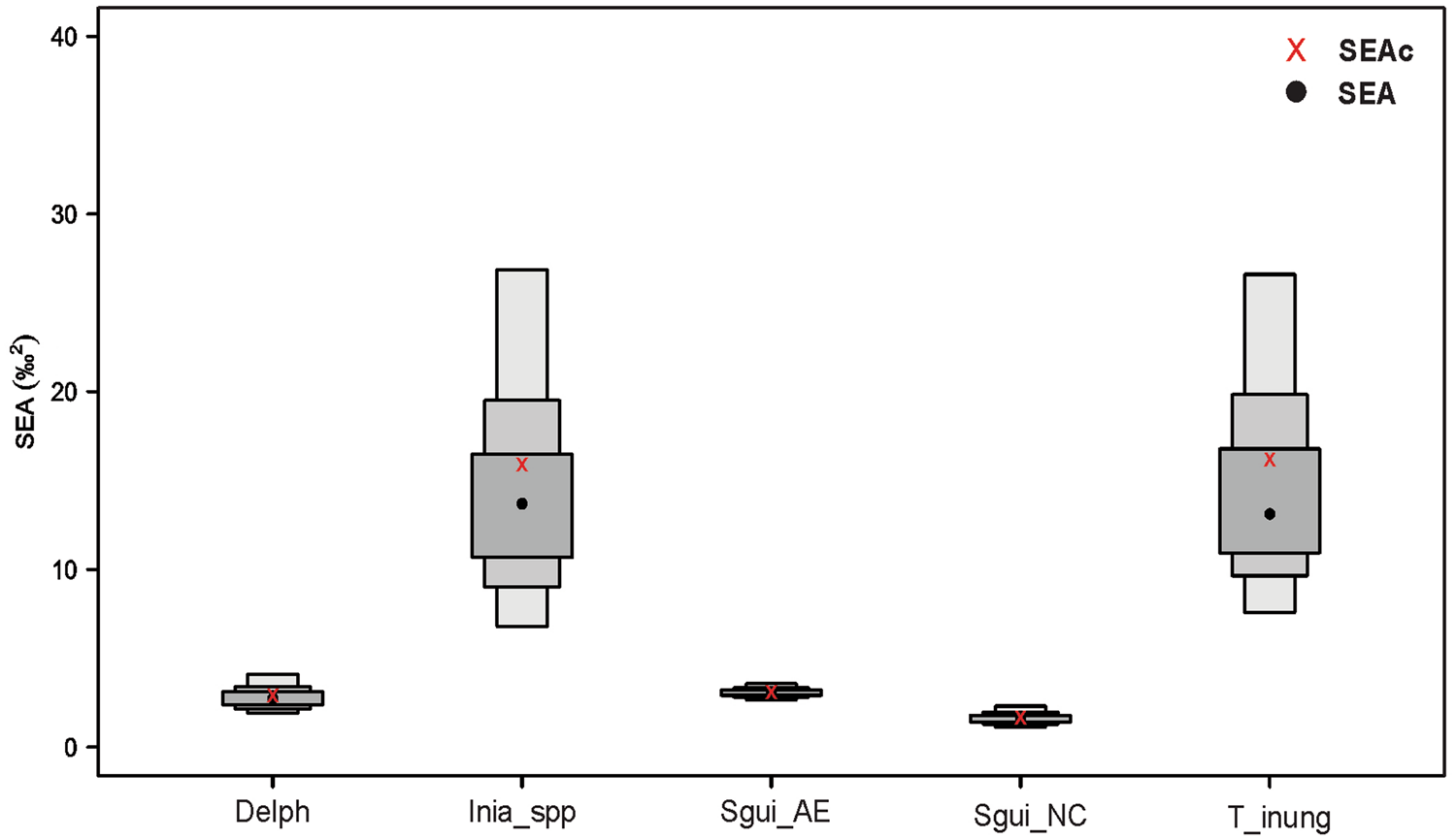
Bayesian results of the variation in δ^13^C and δ^15^N values for Delphinids (Delph), *Inia geoffrensis* and *I. araguaiaensis* (Inia_spp), *Sotalia guianensis* from Amazon estuarine complex (Sgui_AE) and Northeast (Sgui_NC) and *Trichechus inunguis* (T_inung) from Northern and Northeastern Brazil. Measures of Bayesian standard ellipse areas: black circles represent the mode and boxes indicate 50%, 75% and 95% credible intervals from dark grey to light grey, respectively. Red squares indicate the SEAc (SEA corrected for small samples sizes).

Moreover, *T. inunguis* and *Inia* spp. displayed larger SEAc areas compared to that of *S. guianensis* from AE (Table 2, Figs. 4, 5).

**Table 2:** Isotope niche width of five groups of aquatic mammals: *Trichechus inunguis* (T_inung), *Inia geoffrensis and I*. *araguaiaensis* (Inia_spp), Delph (Delphinids) and *Sotalia guianensis* (Sgui_NC and Sgui_AE).

Group	*n*	SEA (‰^2^)	SEAc (‰^2^)
Delph	26	2.8	2.9
Inia_spp	10	14.1	15.9
Sgui_AE	188	3.0	3.1
Sgui_NC	32	1.6	1.7
T_inung	11	14.6	16.2

SEA represents standard ellipse areas and SEAc, standard ellipses adjusted for small sample sizes.

**Figure 6:**
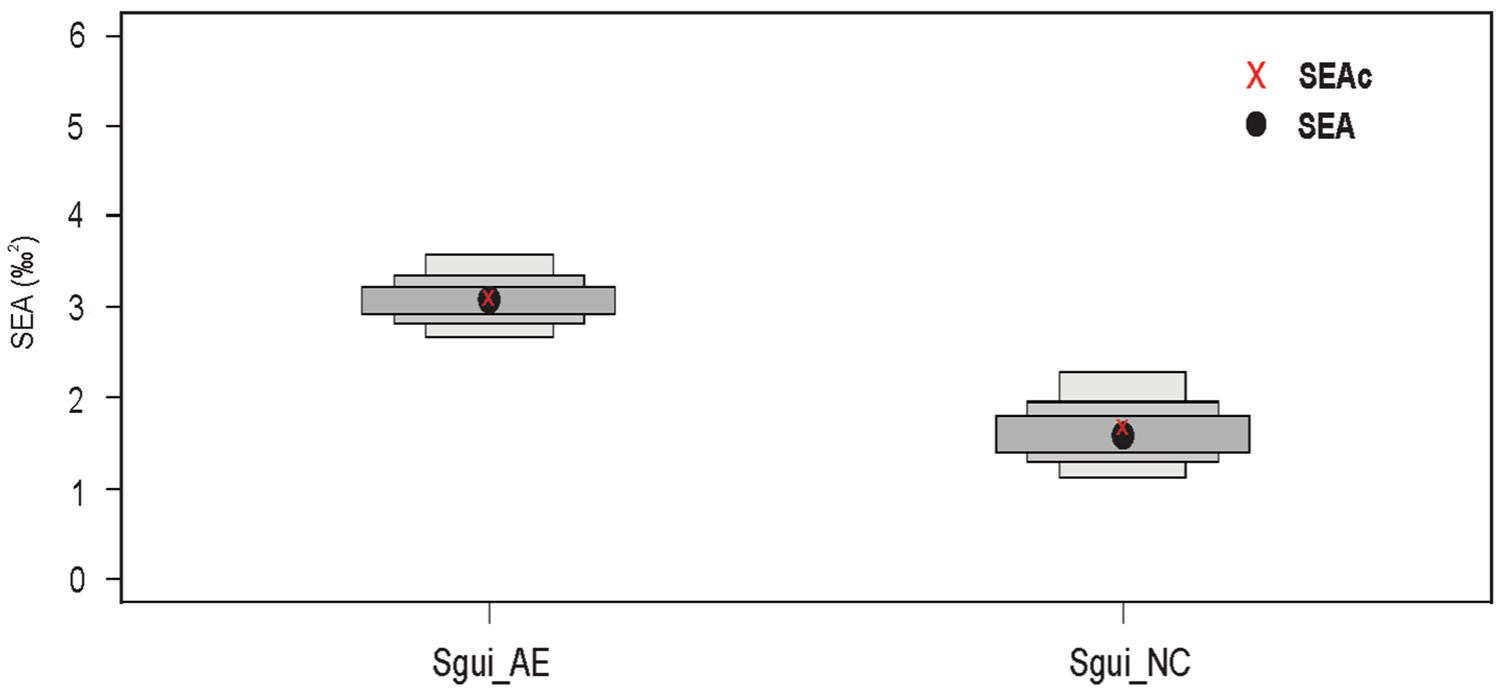
Bayesian results of the variation in δ^13^C and δ^15^N values for the Guiana dolphin *Sotalia guianensis* from the Northeastern coast (NC) and Amazon Estuary and adjacent coastal zone (AE), respectively (Sgui_NC and Sgui_AE). Black circles represent the SEA mode. Boxes indicate 50%, 75% and 95% credible intervals from dark grey to light grey, respectively. Red squares indicate the SEAc (SEA corrected for small samples sizes) of Bayesian standard ellipse areas.

The smallest isotopic niche area was occupied by Sgui_NC, followed by Sgui_AE. *S. guianensis* populations presented a niche area overlap of 68.98% (Fig. 6).

## Discussion

The isotopic composition in bone/teeth that integrate a long-term diet were evaluated for the first time in aquatic mammals from the Amazon estuarine complex and Northeastern Brazil. No isotopic overlap was observed among coexisting species within the Amazon estuarine complex and adjacent coastal areas (AE). Indeed, isotopic niches were highly distinct among *S. guianensis*, *Inia* spp. and *Trichechus inunguis*, indicating strong niche segregation among these sympatric species, at least in Marajó Bay. Among marine delphinids, the carbon isotopic composition in the bone/tooth collagen in offshore species (i.e. *Grampus griseus*, *Globicephala macrorhynchus*) mirrored that of nearshore species (i.e. *Sotalia guianensis*), contradicting the pattern of decreasing δ^13^C values characteristic of many areas around the world^56^ including areas in Southeastern^68^ and Southern Brazil^69^. This pattern can be explained by the huge freshwater discharge into the adjacent oceanic areas that contributes with^13^C-depleted organic material derived from terrestrial and mangrove sources to their dissolved and particulate organic pool^31,70,71^. Therefore, carbon isotopic values in offshore pelagic areas under the influence of the Amazon plume exhibit low δ^13^C values, while higher values are reported for high salinity oceanic waters^72^. The similar carbon isotopic values found in the collagen of estuarine and oceanic species, suggest that the low basal δ^13^C values are transferred up along the food chain resulting in a lower predicting power of this isotope to track the foraging habitat of these species in this peculiar tropical region. Collagen δ^15^N values in the cetacean species analyzed, on the other hand, were generally correspondent to the known trophic habits of the species, where mostly teutophagous delphinid species (i.e. *Grampus griseus*, *Globicephala macrorhynchus*) showed lower values than those with more piscivorous feeding habits (i.e. *Tursiops truncatus*). Among delphinid species, the bottlenose dolphin *Tursiops truncatus* and *Pseudorca crassidens* presented ^13^C- and ^15^N-enriched collagen reflecting their high trophic position in this tropical food web. Both species are considered mainly piscivorous, and as such, they exhibit high trophic levels within pelagic food webs^73−75^. *Steno bredanensis* is considered an offshore species in Southeastern Brazil^76^, although regularly sighted in shallow bays and other coastal areas^77^. Increased stranding events and fishermen reports on *T. truncatus* and *S. bredanensis* reinforces their regular presence off Northern Brazil^39,48^, mostly associated with negative interactions with artisanal fisheries. Indeed, stranding events are considered an important source to access ecology data of marine mammals. The study area is influenced by the water and wind regime of the North Brazil Current (NBC), defined as a major low latitude western boundary current in the Atlantic that transports upper-ocean waters northward across the Equator. NBC does not show significant seasonal variation in the lower-frequency fluctuations in the thermocline layer throughout the year^78^. As such, carcasses are expected to be transported westwards on a regular basis, following trade winds, drifting in a water mass of warm (> 24°C) temperatures. Teutophagous species (e.g., *Globicephala macrorhynchus*, *Peponocephala electra* and *Grampus griseus*) and species that feed on small pelagic fish and squid (e.g., *Lagenodelphis hosei* and *Stenella attenuata*) displayed similar δ^13^C and δ^15^N values, coinciding with their low trophic level predation in offshore areas^79^. So far, no previous isotopic data for *G. macrorhynchus*, *P. electra* and *Physeter macrocephalus* have been reported for Brazilian waters. *P. macrocephalus* samples were the most ^15^N-enriched and ^13^C-depleted compared to the other assessed taxa. Due to the long-time interval integrated in the bone collagen of this long-lived cetacean, these values are possibly averaging their movement between isotopically distinct foodwebs throughout their lives, thus limiting the interpretation of their trophic position within the studied cetacean community. Nevertheless, it is expected that the species occupy high trophic levels within offshore foraging grounds^80,81^ which is supported by the isotopic values in the two mature females and the immature specimens analyzed in this study. Higher nitrogen isotopic values were expected for the coastal *Sotalia guianensis*, regardless of its trophic position, as higher basal nitrogen values were observed in the estuarine waters^82^. Basal nitrogen isotopic values seem to track the main nitrogen sources used by the producers^83^. Indeed, higher δ^15^N values are found within estuarine and nearshore areas dominated by diatoms while lower δ^15^N values, typical of diatom-diazotroph associations and oceanic diazotroph producers, which are reported within the Amazon plume and the adjacent oceanic areas, respectively^32,82,83^. In this context, a higher relative importance of low trophic level prey such as shrimps and squids in the diet of the species^84^ may account for the low nitrogen isotopic values found in this species.

### Niche partitioning

The largest isotopic niche areas were found for *Inia* spp. and *T. inunguis*, mainly resulting from a wide range of δ^13^C values, reflecting higher habitat plasticity, i.e., foraging along a gradient encompassing lowland freshwaters and AE estuarine habitats. Variable carbon isotope values in these aquatic mammals may be the result of a diversity of basal sources found mainly in the Amazon estuarine complex (AE), characterized by the presence of both ^13^C-enriched C_4_ plants (e.g., seagrasses) and low δ^13^C plants, such as mangrove leaves (δ^13^C = − 28.4 ± 0.5‰^85^). Ontogenetic variation were found in manatee diets at the Tapajós and Negro rivers, in the Amazon basin, with a proportional consumption of C4 and C3 plants for lactating females and other adults, respectively^86^. Some potential diet items were identified for both manatees, Amazonian and Antillean, in estuarine Marajó Bay areas (i.e., *Blutaparon portulacoides, Eleocharis geniculata*, *Crenea maritima*), including both C3 and C4 plants^87^.

Vegetation composition and rainfall season could influence the biomass and exposure of seagrass and macroalgae and, consequently, manatee foraging habits^88^. In the Amazon estuarine complex, the local tidal regime probably causes changes in vegetation availability, such as substrate and flood level point differences in size, shape and plant appearance^87^. Manatee movements inside this dynamic habitat can be influenced by food availability, which should vary according to the dry and rainy seasons. The rainy season has a strong influence on the presence of manatees in Marajó Bay^89^.

Until recently, the Amazon River dolphin, *Inia geoffrensis,* was considered endemic to the Amazon and Orinoco basins^90^ while the newly described *I. araguaiaensis* was restricted to the Tocantins-Araguaia River basin^52^. However, Costa et al.^91^ described the presence of *Inia* spp. in estuarine areas of Marajó Island and the eastern Pará coast. Further molecular analyses confirmed the occurrence of both *Inia* species around Marajó Is. and the Curuçá Estuary, extending *I. araguaiaensis* distribution area in nearly 500 km^42^. The small sample size in the present study did not allow for comparisons between the isotopic niche of these two species. Data on one *I. araguaiaensis* collected at Curuçá Estuary was ^13^C-depleted in δ^13^C values, similar to samples from the AL sector, suggesting that it probably fed in a ^13^C-depleted food web (e.g., mangrove)^85^ and moved to estuarine areas. Prey availability could determine this species movements and might affect species distribution^92,93^. Although *I. geoffrensis* and *I. araguaiaensis* are considered exclusively freshwater species^52,90^, the findings reported herein reinforce that the use of estuarine areas is not circumstantial but rather reflects their occupancy in these environments for active foraging.

Although *S. guianensis* and *Inia* spp. are considered piscivorous species^90,94,95^ no isotopic niche overlap was observed, indicating spatial and trophic partitioning, at least in Marajó Bay. Studies on the trophic ecology of *I. geoffrensis* are scarce, although the analysis of stomach contents revealed at least forty-three fish species^90^. However, no studies describing the species’ diet in flooded areas of the eastern Amazon or in the Amazon estuarine complex are available to date.

Isotopic values indicate that *Inia* spp. forage at higher trophic levels than *S. guianensis*. Morphological *Inia* characters (e.g., lateral mobility) probably allow individuals to explore areas with dense vegetation in floodplains (i.e., *várzea*) and flooded forests (i.e., *igapó*)^96^. In the Central Amazon *I. geoffrensis* and *S. fluviatilis* have been observed foraging in extensive floodplain areas around main rivers (Solimões and Amazonas). In this region, the flood cycle impact can determine both habitat and prey availability^92^. Differences in habitat use, foraging strategies and prey availability, as well as consumption of prey belonging to different trophic guilds, could reduce inter-specific competition between *Inia* and *Sotalia* in sympatric areas.

### Ecological *Sotalia guianensis* stocks

At least six management units for *S. guianensis* have been suggested alongshore Brazil (Pará, Ceará, Rio Grande do Norte, Bahia, Espírito Santo and South-Southeastern area) through the use of molecular markers, evidencing a strong population structure^51^. Other ecological parameters have been used to differentiate ecological stocks. Different geographic areas (Espírito Santo, north and south Rio de Janeiro and São Paulo) have been recognized mainly for cranial and feeding apparatuses variables^97^. Cranial morphometry exhibited a latitudinal growth pattern from North to South, i.e., specimens from São Paulo had smaller skulls. Skull analyses from the north, northeast and southeast have been demonstrated a complete separation between Pará and Rio de Janeiro populations, and a partial separation between Pará, Maranhão and Piauí^98^. Analyses of the periotic-tympanic bone complex of three Brazilian regions also reported geographic variations^99^.

In order to evaluate ecological *S. guianensis* stocks, Botta^100^ analyzed stable isotopes in Guiana dolphin teeth from the Amazon River estuary, Ceará, Espírito Santo, northern Rio de Janeiro, southern São Paulo and northern Paraná and northern Santa Catarina. Four groups were recognized, indicating that SIA is a powerful tool to confirm ecological stock differences.

Significant differences in isotopic carbon composition were found for *S. guianensis* from AE and NC in the present study, although with similar nitrogen isotope values. Sgui_AE bone collagen samples were more depleted in ^13^C than those from Sgui_NC. Dolphins from AE occupy a broader isotopic niche area, indicating higher trophic plasticity. Groups in this sector probably have a larger availability of more diverse prey associated to freshwater habitats^101,102^ and carbon source variability due to the huge influence of the Amazon River and Pará River estuary (*i.e*. Marajó Bay). Individuals from Sgui_NC occupy a narrower isotopic niche area, probably resulting from the association of a greater consumption of marine fishes, as identified in stomach contents of individuals from that population^102^.

In summary, this study provided a first attempt to evidence trophic relationships and resource partitioning among aquatic mammals from the Amazon estuarine complex and its adjacent coastal zones. The obtained SIA results allowed for insights on habitat use and the trophic ecology of marine mammals inhabiting different oceanographic regions off Northern/Northeastern Brazil. Furthermore, δ^13^C and δ^15^N values in *S. guianensis* inhabiting two coastal regions (AE and NC) indicated an ecological distinction between the populations. These findings provide novel data on the stable isotope composition for these threatened cetaceans and sirenian species in this large section of Brazilian coast.

## Supplementary information

Supplementary file1 (DOCX 16 kb)
