# Supplementary material for: Resource partitioning among stranded aquatic mammals from Amazon and Northeastern coast of Brazil revealed through Carbon and Nitrogen Stable Isotopes
Source: Sci Rep. 2020 Jul 30;10:12897. doi: 10.1038/s41598-020-69516-8 (PMC7393136; doi:10.1038/s41598-020-69516-8)
Supplement: Supplementary file 1 — Supplementary file1 (DOCX 16 kb) [file 41598_2020_69516_MOESM1_ESM.docx]

**Resource partitioning among stranded aquatic mammals from Amazon and Northeastern coast of Brazil revealed through Carbon and Nitrogen Stable Isotopes**

**Alexandra F. Costa^1^, Silvina Botta^2^, Salvatore Siciliano^3^, Tommaso Giarrizzo^1^**

1 Núcleo de Ecologia Aquática e Pesca da Amazônia, Universidade Federal do Pará, Av. Perimetral 2561, Terra Firme, 66040-170, Belém, PA, Brazil

2 Laboratório de Ecologia e Conservação da Megafauna Marinha, Instituto de Oceanografia, Universidade Federal do Rio Grande - FURG, 96203-900 Rio Grande, RS, Brazil

3 Laboratório de Biodiversidade, Instituto Oswaldo Cruz/Fiocruz, Pavilhão Mourisco, sala 217, Manguinhos, Av. Brasil, 4365 - Manguinhos, 21040-900, Rio de Janeiro, RJ, Brazil

Correspondence to Alexandra F. Costa (email: alexandrafernandescosta@gmail.com)

**Table S1**. Species of aquatic mammals analyzed during this study from Amazon Estuary and adjacent area, Northeastern coast of Brazil and their use habitats, evidence of occurrence and references used to characterize species.

| Species | Common name | Habitat | Data evidence | Reference |
| --- | --- | --- | --- | --- |
| *Trichechus inunguis* | Amazonian manatee | Freshwater/estuarine^@^ | Stranding, sighting, rescue, SIA | Crema et al 2019; Siciliano et al. 2008; Carvalho et al. (*in review*), this study |
| *Inia geoffrensis* | Amazon river dolphin | Freshwater/estuarine^@^ | Stranding, molecular data | Siciliano et al 2016; Costa et al. 2013; Siciliano et al. 2008, this study |
| *Inia araguaiaensis* | Araguaian boto | Freshwater/estuarine^@^ | Stranding, molecular data | Siciliano et al 2016; Hrbek et al 2014; Costa et al. 2013, this study |
| *Sotalia guianensis*  Amazon Estuary | Guiana dolphin | Coastal^%^ | Stranding, sighting, stomach contents, SIA | Costa et al. 2017; Vieira, 2014; Botta et al. 2012; Botta, 2011; Emin-Lima et al 2010; Siciliano et al. 2008; this study |
| *Sotalia guianensis*  Northeastern Coast | Guiana dolphin | Coastal^%^ | Stranding, sighting, stomach contents, SIA | Costa et al. 2017; Siciliano et al. 2008; this study |
| *Steno bredanensis* | Rough-toothed dolphin | Coastal^%^ | Stranding, sighting, SIA | Costa et al. 2017; Ramos et al 2009, Siciliano et al. 2008; this study |
| *Tursiops truncatus* | Bottlenose dolphin | Coastal^%^ | Stranding, sighting, SIA | Costa et al. 2017; Siciliano et al. 2008, this study |
| *Stenella attenuata* | Pantropical spotted dolphin | Continental shelf* | Stranding, sighting, SIA | Costa et al. 2017; GEMAM, unpublished data, this study |
| *Delphinus* sp. | Common dolphin | Continental shelf* | Stranding, sighting, SIA | Costa et al. 2017, this study |
| *Globicephala macrorhynchus* | Short-finned pilot whale | Continental shelf* | Stranding, sighting, SIA | Costa et al. 2017; Siciliano et al. 2008; this study |
| *Lagenodelphis hosei* | Fraser's dolphin | Continental shelf* | Stranding, SIA | Costa et al. 2017, this study |
| *Grampus griseus* | Risso's dolphin | Continental shelf* | Stranding, sighting, SIA | Costa et al. 2017; Siciliano et al. 2008, this study |
| *Peponocephala electra* | Melon-headed whale | Continental shelf* | Stranding, SIA | Costa et al. 2017, this study |
| *Pseudorca crassidens* | False killer whale | Continental shelf* | Stranding, SIA | Costa et al. 2017, this study |
| *Physeter macrocephalus* | Sperm whale | Oceanic^#^ | Stranding, sighting, SIA | Costa et al. 2017; Siciliano et al. 2008; this study |

Large pelagic cetaceans (Oceanic^#^); Small pelagic cetaceans (Continental shelf ^*^); Small coastal cetaceans (Coastal^%^); Freshwater/estuarine^@^
